# Current status of pathogen handling in European laboratories: focus on viral inactivation process

**DOI:** 10.3389/fbioe.2024.1422553

**Published:** 2024-06-07

**Authors:** Boris Pastorino, Franck Touret, Magali Gilles, Xavier De Lamballerie, Remi N. Charrel

**Affiliations:** ^1^ Unité des Virus Émergents (UVE: Aix-Marseille Univ, Università di Corsica, IRD 190, Inserm 1207, IRBA), Marseille, France; ^2^ Laboratoire des Infections Virales Aigues et Tropicales, AP-HM Hôpitaux Universitaires de Marseille, Marseille, France; ^3^ LE Service de Prévention du Risque Infectieux (LESPRI), CLIN AP-HM Hôpitaux Universitaires de Marseille, Marseille, France

**Keywords:** virus inactivation, biosafety, inactivation guidelines, European laboratories, LAIS

## Abstract

For handling safely infectious agents, European laboratories must comply with specific EC Directives, national regulations and recommendations from the World Health Organization (WHO). To prevent laboratory acquired infections (LAIs) and pathogens dissemination, a key biosafety rule requires that any infectious material (clinical specimens or research samples) manipulated outside a biosafety cabinet (BSC) must be inactivated unless the lack of infectivity is proven. This inactivation process is a crucial step for biosafety and must be guided by a rigorous experimental qualification and validation procedure. However, for diagnostic or research laboratories, this process is not harmonized with common standard operation procedures (SOPs) but based on individual risk assessment and general international guidelines which can pose problems in emergency situations such as major outbreaks or pandemics. This review focuses on viral inactivation method, outlining the current regulatory framework, its limitations and a number of ways in which biosafety can be improved.

## Regulations and practices for handling pathogens

Prevention of exposure to biological agents during occupational activities is ruled by European Union Framework Directives 89/391/EEC, 2000/54/EC1 and the 2019/1833 and 2020/739 amendments. Several member states have transposed these directives into national legislation (EASH, 2021; Bielecka and Mohammadi, 2014). Biosafety regulations aim at protecting laboratory workers against risks associated with occupational exposure to biological agents. Due to varying adaptation of EU directives and different approaches to biosafety around the world, several national agencies have provided guidelines covering the use of biological agents (Table 1). For each EU institution and unlike the U.S. Federal Select Agent Program, these guidelines are not legally binding but must be implemented in correlation with the biosafety regulation of national and international policies. They include a set of rules and procedures to be used and enforced by personnel working in the facilities where pathogens are handled. These biosafety guidelines address i) a bio-risk assessment for each agent, ii) specific biosafety measures covering the code of practice, iii) the design of biocontainment laboratories together with entry and exit procedures, iv) required equipments and rules for maintenance, v) medical surveillance of personnels based on occupational risk, vi) staff training, vii) safe handling of chemicals. For each laboratory, the development of biosafety guidelines is part of the overall quality management systems implementation and allows biosafety adaptation to the environment and working practices.

**TABLE 1:** Selected regulatory agencies and guidelines related to methods for virus removal/inactivation.

Regulatory agencies	Biosafety guidelines
The European Committee for Standardisation (CEN)	- The European tiered approach for virucidal efficacy testing–rationale for rapidly selecting disinfectants against emerging and re-emerging viral diseases (2020)
The European Centre for Disease Prevention and Control (ECDC)	- Biosafety Europe-Coordination, Harmonisation and Exchange of Biosafety and Biosecurity Practices within a pan-European Network
	- Laboratory Support for COVID-19 in the EU/EEA
The European Medicines Agency (EMA)	- ICH Guideline Q5A (R2) on viral safety evaluation of biotechnology products derived from cell lines of human or animal origin (2022)
	- Note for guidance on virus validation studies: the design, contribution and interpretation of studies validating the inactivation and removal of viruses (1996)
European Chemical Agency (ECA)	Guidance on the Biocidal Products Regulation Volume II: Efficacy Parts B + C: Assessment and Evaluation Version 5.0, November 2022
Health and Safety Executive (UK)	- Infection at Work: Controlling the Risks
	- Biological agents: Managing the Risks in Laboratories and Healthcare Premises
Public Health England	COVID-19: safe handling and processing for samples in laboratories Updated 29 March 2021
The Centers for Disease Control and Prevention (CDC)	- Biosafety in Microbiological and Biomedical Laboratories (BMBL) 6th Edition
The U.S. National Institutes of Health (NIH)	
The FDA’s Center for Biologics Evaluation and Research (CBER)	Points to Consider in the Manufacture and Testing of Monoclonal Antibody Products for Human Use, CBER, 1997
The World Health Organization (WHO)	WHO Laboratory Biosafety Manual, 4th edition
The Centers for Disease Control and Prevention (CDC)	Guidance on the Inactivation or Removal of Select Agents and Toxins for Future Use
The U.S. Department of Agriculture (USDA)	

To summarize the EU regulatory framework for handling pathogens based on WHO guideline, i) the risk group classification is the legal guide to the minimum containment level and measures required to work with a biological agent but ii) a risk assessment must also be performed, and appropriate mitigation measures must be identified and implemented as part of an institution’s comprehensive biosafety program before laboratory work can be autorized. Accordingly, the laboratory director is ultimately responsible to ensure that risk is assessed, safety measures and practices are reviewed routinely and revised when necessary (WHO, 2020b).

Dispensations from containment measures are regulated and various domestic and international public health organizations have published guidelines on sample handling for viral detection, particularly for SARS-CoV-2. These guidelines generally state that non-propagative diagnostic laboratory work can be conducted in BSL-2 facilities, and that initial sample processing (before inactivation) should take place inside a biosafety cabinet (BSC) (WHO, 2020a; CDC, 2021).

But in any case, and to ensure biosafety, the handling of infectious pathogens, whether for diagnostic or research purposes, often requires an inactivation step.

## Virus inactivation in laboratory settings: status and limitations

The inactivation step aims at minimizing the risk associated with the handling of an infectious sample during diagnostic or research procedures (virus growth and isolation), i.e., the risk of direct contamination of exposed workers and the risk of indirect exposure through infectious fomites. Biosafety guidelines recommend that procedures for chemical or physical inactivation are validated or verified in-house (at each institution) to guarantee complete inactivation. This process is legally required only for specific pathogens in the U.S. or France (respectively for select agents or MOTs). In fact, the need for local qualification has been decided because a large number of parameters can affect the efficacy of a technical protocol (physicochemical characteristics of the virus, viral load, cell number, volume, sample composition, exposure time, temperature, concentration of the inactivating chemical, limit of detection of the testing modality, etc.).

Inactivation methods are highly reliable and can be reproducible across viral families, however demonstration of inactivation is based on culturing or growth evaluation procedures that are not standardized and can differ between laboratories (Davies et al., 2021; Widera et al., 2021).

In-house validation is acceptable whether i) it is strictly identical to a published protocol, ii) it is using the exact same conditions and reagents of a commonly recognized protocol, or iii) it is an institution proprietary protocol with technical material and method section allowing reproducibility. Consequently, implementing and validating a protocol for virus inactivation is more complex than it appears for a number of reasons among which we can list:

- The absence of specific standards or harmonized guidelines for clinical or research infectious samples
- The lack of consultation and coordination between laboratories for sharing protocols
- The need for accessing a high containment laboratory to carry testing assays on replicative BSL3/4 viruses or clinical samples spiked with such agents.
- The need to assess the impact of inactivation methods on assay read-outs (sensitivity/reproducibility)
- The large number of different chemicals products used in laboratories for inactivation (extraction buffers, formaldehyde solutions…).
- The lack of information on widely used marketed buffers present in pathogen diagnostic kits (compositions, virucidal activity)

European standards (EN) and testing method are available only for chemical inactivation not for thermal or physical procedures despite the two latter are widely used (Eggers et al., 2021; AFNOR, 2019). Chemical inactivation process employs the use of denaturing chemicals (alcohols, aldehydes, detergents) to denature proteins and render the agent(s) inactive. Examples of chemicals used for inactivation include Glutaraldehyde, formaldehyde, Paraformaldehyde; Triton X-100; Sodium Dodecyl Sulfate; Phenol/Chloroform/Isoamyl alcohol; Ethanol, Diethyl ether or Acetone. Some limitations exist for diagnostic or research activities as over-aggressive chemical treatment can removed or damage characteristics of interest in the material (e.g., DNA/RNA integrity for PCR; peptides, proteins, and antibodies for ELISA, etc.). Moreover, the EN-14476 chemical standard is validated for an infectious titer reduction of at least 4 log10 even if the sample remains infectious. Consequently, this norm is not suitable for ensuring the biosafety of exposed workers. This point is particularly problematic for many viruses where the infectious dose is low or is undefined.

In medical virology laboratories, guaranteeing a complete inactivation applicable to clinical specimens is very important due to the variety of specimens (e.g., whole blood, serum, plasma, stool, nasopharyngeal aspiration, swabs …). Moreover, diagnostic laboratories are increasingly equipped for molecular biology and for serology with random access automates; such equipment cannot guarantee airborne prevention and need to be fueled with previously inactivated clinical samples in either primary or secondary tubes.

Since EN-14885 and EN-14476 are not fully adapted to purposes described here, it is necessary for draw up guidelines to harmonize virus inactivation protocols. In the meantime, when specific standard test methods are not available, the biocidal product regulatory guidelines published by the European Chemicals Agency (ECHA) recommend using guidelines from other national or international standardization bodies (European Chemicals Agency, 2022).

For example, with regards to biopharmaceutical products, the design of virus clearance studies is the subject of numerous regulatory guidelines that could be useful to develop a relevant virus removal/inactivation process (US Food and Druck, 2022; European Medicine Agency, 1996).

## Process to guarantee biosafety

**FIGURE 1:**
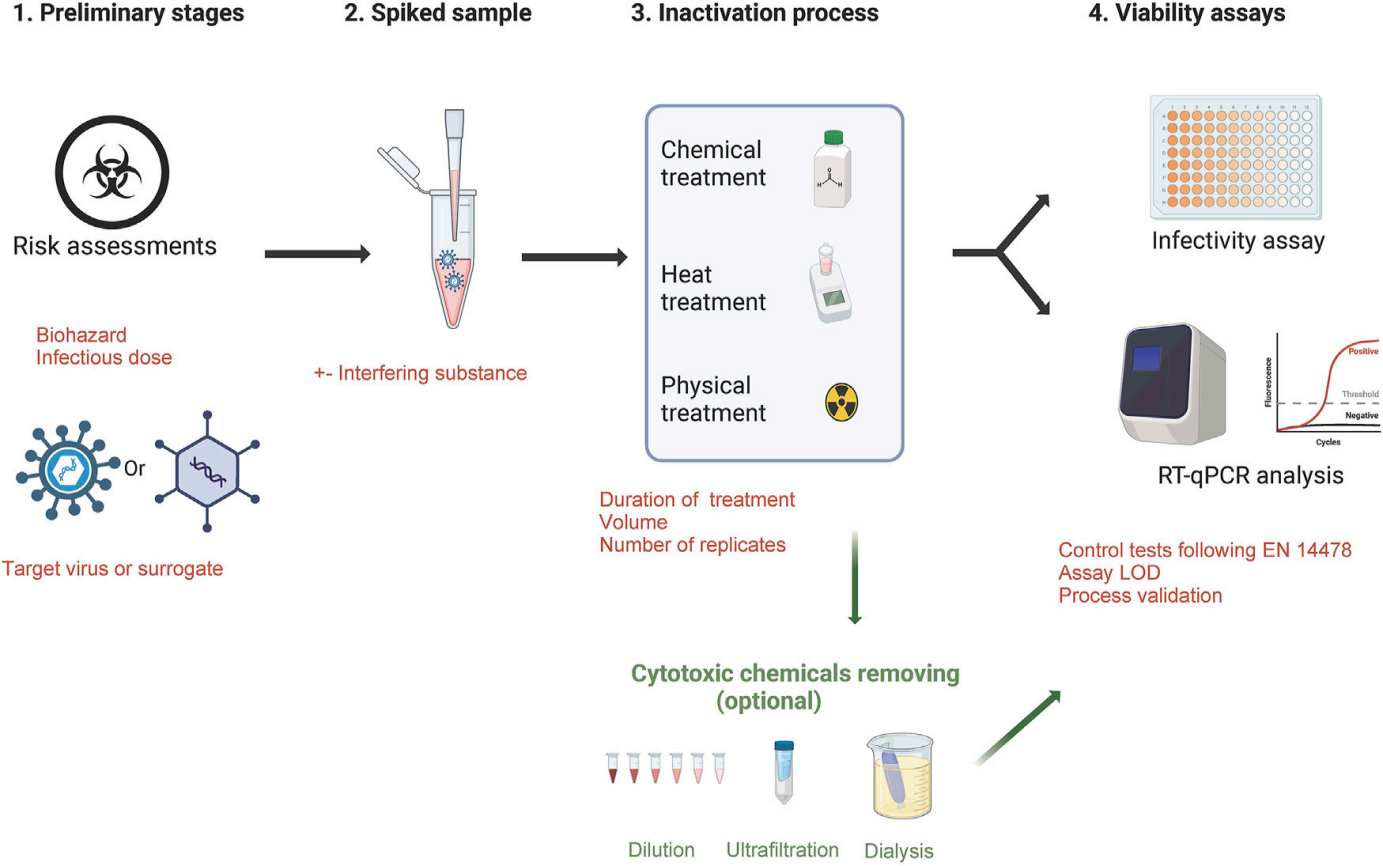
Essential steps to validate a virus inactivation method.

For European diagnostic and research laboratories, the validation of inactivation methods is not standardized or legally regulated (except for the MOTs law in France) and must be confirmed by data generated from a viability testing protocol. To build a robust process ensuring an optimal biosafety, some essential points are required (Figure 1):

- Assessing the quality of risk assessments for the identified hazard/define the viral load to be used for experiments and set-up the acceptable residual infectivity
- For highly pathogenic viruses (BSL-4 list) or for viruses that are difficult to grow in cell monolayers, evaluate the use of a surrogate virus and the criteria to designate one
- Virus and reference controls must be included in the protocol
- Create a spiked sample with virus titer largely above the expected inactivation effectiveness of the tested protocol in order to provide a quantified efficacy
- define the number of replicates
- Assess the limit of detection of the testing protocol
- consider the presence of residual chemicals in samples could skew the results of the viability test causing false negatives. In this case, a step to remove cytotoxic substances is required (washing of cells with buffer, neutralization of the cytotoxic component, elimination via ultrafiltration spin columns, dialysis, dilution)
- Suppression or interference controls are also required by i) checking the efficiency of the neutralising method in suppressing the virucidal activity of the test product after the required exposure time, ii) checking the susceptibility of infection in cells is not influenced negatively by the test product

Moreover, to reach a complete inactivation for highly pathogenic or high infectivity samples, several studies and WHO guidelines suggest the combination of inactivating techniques including at least two different modes of action (Elveborg et al., 2022).

Ideally, an inactivation procedure must be tested with the targeted virus; however, in certain circumstances a surrogate virus can replace the targeted virus. The surrogate virus must present biological characteristics and properties that are as close as possible to the target virus. Once cell non-infectivity has been demonstrated, and the record requirements are met, the material is no longer subject to biosafety guidelines recommendation or specific regulations (select agents or MOTs). It can be moved to a laboratory displaying a lower level of biocontainment (CDC-USDA, 2018).

The effectiveness of inactivation protocols can be optimized by drawing on the experience of external quality assessment (EQA) programs. These programs should be adapted to viral inactivation in the context of research laboratories. It is known that the key instrument to ensure the highest possible standard of technical analyses (PCR, ELISA, Neutralization assay, LC/MS…) and to achieve a harmonization of their results is an external quality assessment (EQA). For viral detection, these types of actions have proven their effectiveness to identify problems and verify laboratory performance against other laboratories using external agencies (Sung et al., 2020; Mögling et al., 2022). Relying on national networks of reference laboratories, viral inactivation protocols could be developed and validated before use in other laboratories. Titrated viral standards (wild type strain or pertinent surrogates) could be distributed in the form of ring testing to confirm that the inactivation protocol provides the expected results in particular on the basis of effectiveness (titer reduction), replicability and target integrity for further analysis.

## Ways for future improvement

European research laboratories and clinical laboratories have not yet adopted a uniform approach for the inactivation of infectious samples. Regulations still require that procedures used to demonstrate the chemical or physical inactivation of agents must be validated or verified in-house (under the sole responsibility of each institution) to confirm inactivation (European Medicine Agency, 1996; CDC-USDA, 2018; AFNOR, 2019; Sung et al., 2020; CDC, 2021; Davies et al., 2021; Eggers et al., 2021; Widera et al., 2021; Elveborg et al., 2022; European Chemicals Agency, 2022; US Food and Druck, 2022). Consequently, there are many studies or guidelines describing specific protocols for chemical or physical inactivation of viruses. In practice, this regulatory framework does not harmonize practices or guarantee their effectiveness as demonstrated by recent viral outbreaks and the need for systematically published new protocols to protect laboratory workers (Quéromès et al., 2023).

These problems could be easily solved by setting up common standard operating procedures for virus inactivation methods and some measures could speed up such implementation:

The creation of norms or standards adapted to the laboratory inactivation process that will improve and clarify biological safety (by analogy with the EN ISO 22442 standard), the development of a specific European standardized test approach by the European Committee for Standardization (CEN), the marketing of virucide-certified buffers for pathogen diagnostic reagents, the inclusion in regulatory guidelines of a clear and consistent definition of inactivation as well as a precise description of the validation process.

In the same way and for molecular screening, the development of a large scale process for heat inactivation of clinical samples prior to laboratory processing is a promising method to improves operator safety (Delpuech et al., 2022).

## Conclusion

Diagnostics and research of high-risk pathogens in lower biosafety environments depends on the availability of safe and efficacious inactivation methods. For that, the use of CEN test methods is highly recommended however due to the contrasting literature regarding virus inactivation and no specific regulations to validate the method, a case-by-case assessment of different inactivation protocols is essential to prevent laboratory-acquired infections. In fact, various laboratory methods have been developed for virus inactivation activity testing that differ in their design and experimental details. All are based on the principle of adding a test inoculum to a targeted sample or using biological material removed from virus infected animals, applying the inactivation method and then measuring pathogen infectivity. To guarantee biosafety, some standard tests (e.g., EN tests 14485/EN 14476/EN ISO 22442) as well as existing guidelines contain examples of appropriate reports, which should be used as template to validate relevant inactivation process for pathogen handling in European laboratories.

## Data Availability

The raw data supporting the conclusion of this article will be made available by the authors, without undue reservation.
